# *prox1b* Activity Is Essential in Zebrafish Lymphangiogenesis

**DOI:** 10.1371/journal.pone.0013170

**Published:** 2010-10-18

**Authors:** Luca Del Giacco, Anna Pistocchi, Anna Ghilardi

**Affiliations:** 1 Department of Biology, Università degli Studi di Milano, Milan, Italy; 2 Division of Regenerative Medicine, San Raffaele Scientific Institute, Milan, Italy; Louisiana State University, United States of America

## Abstract

**Background:**

The lymphatic vascular system, draining interstitial fluids from most tissues and organs, exerts crucial functions in several physiological and pathological processes. Lymphatic system development depends on *Prox1*, the first marker to be expressed in the endothelial cells of the cardinal vein from where lymph vessels originate. *Prox1* ortholog in the optically clear, easily manipulated zebrafish model has been previously isolated and its contribution to lymphangiogenesis has been clarified. Because of a round of genome duplication occurred at the base of teleosts radiation, several zebrafish genes have been retained in duplicate through evolution. We investigated for the presence of additional *prox1* genes and determined their role in zebrafish lymphangiogenesis.

**Methodology/Principal Findings:**

We isolated a second ortholog, named *prox1b*, and analyzed its expression during development by whole mount *in situ* hybridization (WISH). We detected strong *prox1b* expression in the endothelium of the posterior cardinal vein (PCV) from where lymphatic precursors originate. To analyze *prox1b* involvement in lymphangiogenesis we utilized the fli1:GFP transgenics and followed the formation of the toracic duct (TD), the primary lymph vessel in fish, after *prox1b* knockdown. Our findings clearly demonstrated that the absence of *prox1b* activity severely hampers the formation of the TD.

**Conclusions/Significance:**

This work provides substantial progress toward the understanding of zebrafish lymphangiogenesis. In light of the features shared by the lymphatic systems of zebrafish and higher vertebrates, the establishment of such lymphatic model will provide a powerful tool to study, for instance, disorders of body fluid homeostasis, inflammation and cancer metastasis, and may ultimately contribute to novel therapies.

## Introduction

The lymphatic system drains lymph away from tissues and organs back to the bloodstream, and plays a main role in the immune response and fat absorption. In addition, the lymphatic system is implicated in inflammation processes as well as cancer metastasis.

*Prox1* homeogene, the vertebrate homolog of *prospero* in *D. melanogaster* [1], regulates cell proliferation, fate determination and differentiation during embryonic development, and triggers the molecular program leading to the formation of the lymphatic system. Indeed, ablation of *Prox1* in mouse, Xenopus, and zebrafish impaired the formation of lymph vessels [2], [3], [4], [5], [6], [7].

About 350 million years ago, a round of genome duplication occurred at the base of teleosts radiation resulting in two copies of all genes. Some of such copies have been retained in duplicate through evolution [8]. In view of this evidence, we searched for additional *Prox1* orthologs in zebrafish. Interestingly, while we were conducting the experiments reported in this work, a paper describing the isolation and the expression patterns of two *prox1* genes in the fish medaka has been published [9].

Here, we isolated a second zebrafish ortholog, named *prox1b* (to distinguish it from the first identified one, from now on designated *prox1a*), and showed that *prox1b* knockdown impedes the formation of the toracic duct (TD), the primary fish lymph vessel [10].

## Results and Discussion

### *prox1b* cloning and gene structure

**Figure 1:**
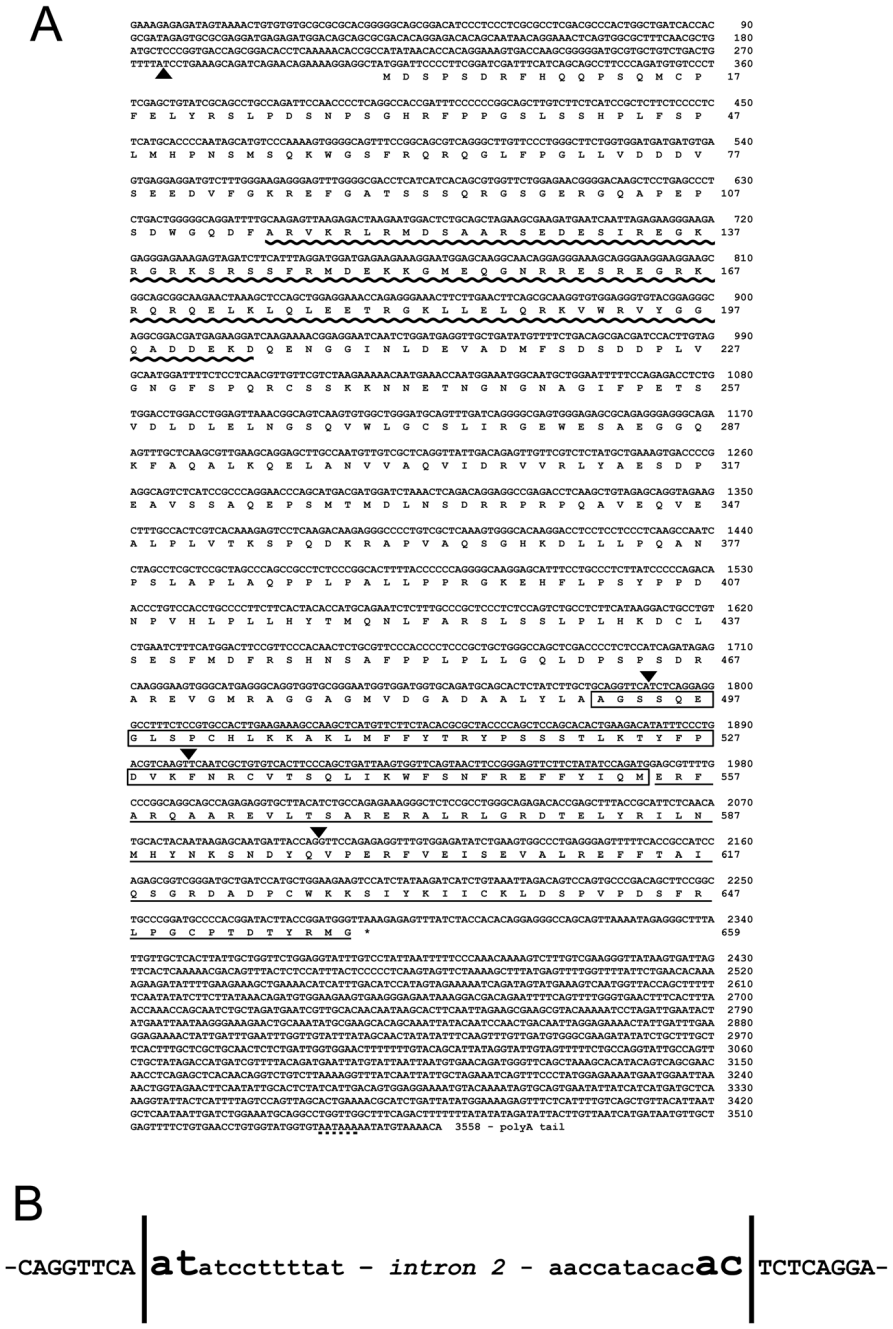
Nucleotide and deduced amino acid sequences of zebrafish *prox1b*. (A) arrowheads show the positions of the introns. The asterisk indicates the stop codon at the end of the open reading frame. A dotted line marks the polyadenylation site. The wave and solid lines mark PD1 and PD2, respectively. The homeodomain is boxed. The sequence has been submitted to the GenBank/EMBL database under accession number FJ544314. (B) *prox1b* contains the U12-dependent intron (intron 2), characterized by the unusual AT/AC splice sites, located at the beginning of the homeobox.

Blast analysis of the ENSEMBL zebrafish assembly version 6 (Zv6) using zebrafish *prox1a* full-length cDNA sequence returned two positive hits on chromosome 17, corresponding to the previously characterized *prox1a* [11], and *prox2* [12] genes, and one hit on chromosome 7, this last one mapping at chromosome location 19,523,485 – 19,537,485 and never reported before. The 3,558-bp mRNA sequence is identified by the GenBank accession number FJ544314 (Figure 1). The encoded protein is related to the prospero transcription factors family, being characterized by the atypical homeodomain and two prospero domains, PD1 and PD2 (Figure 1A). This protein represents the second zebrafish ortholog of Prox1, producing significant alignments (35% of identity) with all the Prox1 present in the GenBank database, including zebrafish prox1a. *prox1b* coding region is interrupted by three introns, and an additional one is located in the 5′ UTR (Figure 1A), indicating that the structure of the gene has been evolutionarily conserved from zebrafish to mammals. [12], [13]. However, zebrafish *prox1b* gene does not show synteny with mammalian *Prox1*, as for the recently identified medaka *prox1b* gene [9]. Analogously to all the vertebrate *Prox* genes, as well as *Drosophila prospero*, *prox1b* contains the U12-dependent intron (intron 2), characterized by the unusual AT/AC splice sites, located at the beginning of the homeobox (Figure 1B) [12], [13].

### Role of *prox1b* in zebrafish lymphangiogenesis

**Figure 2:**
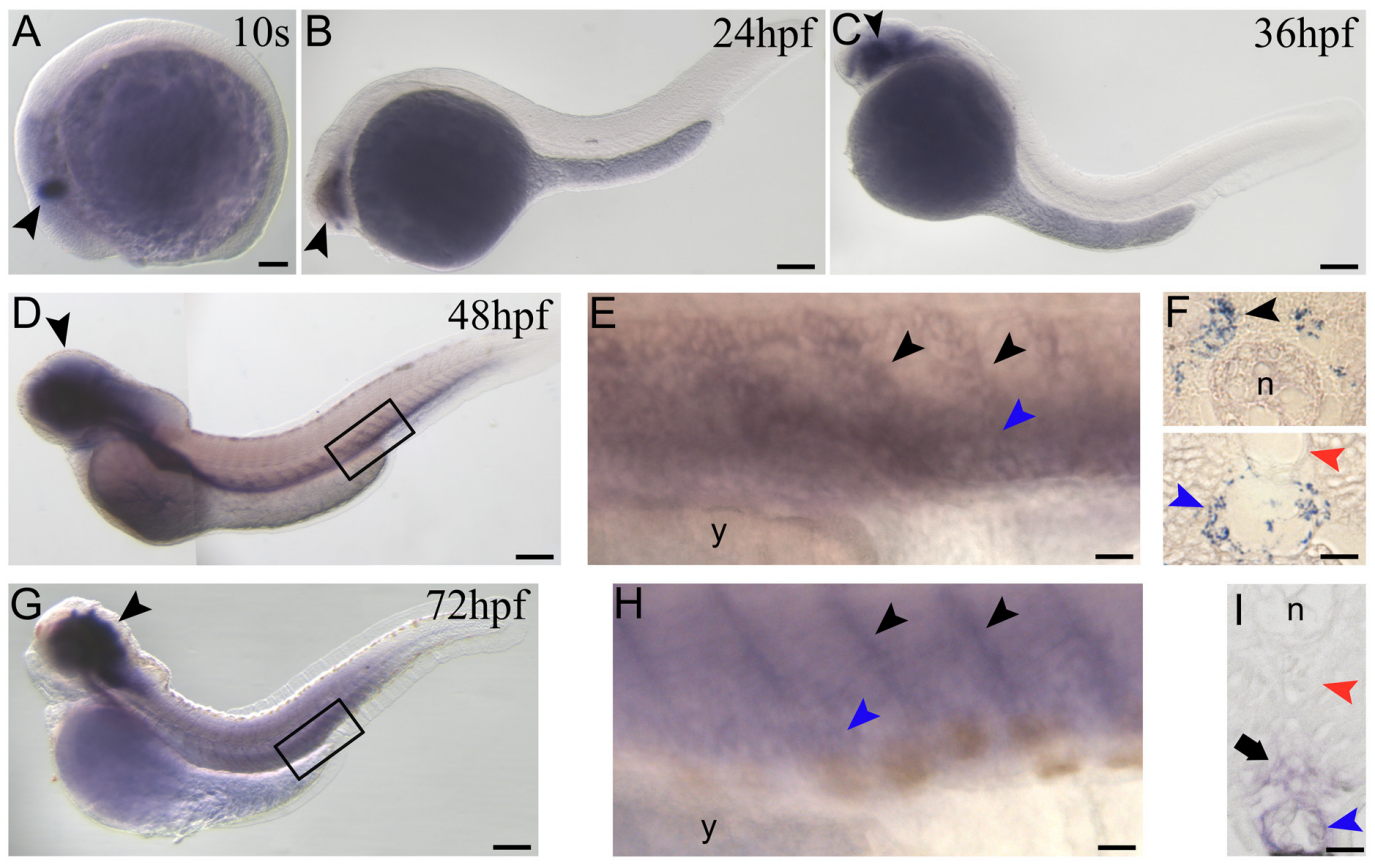
*prox1b* embryonic expression pattern analyzed by *in-situ* hybridization. (A) the first *prox1b* signal appears at 10 somite (s) stage in the central nervous system (CNS) (black arrowhead). (B) at 24hpf and (C) 36 hpf *prox1b* expression continues to be restricted to the CNS of the embryos (black arrowheads). (D) at 48 hpf *prox1b* mRNA is still detectable in the CNS, and is now expressed in the posterior cardinal vein (PCV) and the sprouts emanating from this vessel into the intersegmental space (black box). (E) magnification of the region boxed in D; PCV (blue arrowhead) and vein sprouts (black arrowheads) are indicated. (F) cross sections of 48 hpf-*prox1b*-hybridized embryos. (F, bottom) *prox1b* is expressed in the endothelial cells of the vein wall (blue arrowhead), and not in the dorsal aorta (DA) (red arrowhead). (F, top) *prox1b* signal is also visible on both sides of the trunk (black arrowhead), adjacent to the notochord (n). (G) at 72 hpf *prox1b* expression pattern is comparable to the 48 hpf stage, being the signal detectable in the CNS (black arrowhead), in the PCV and the sprouts emanating from the PCV (black box). (H) magnification of the region boxed in G; PCV (blue arrowhead) and vein sprouts (black arrowheads) are indicated. (I) cross section of a 72 hpf-*prox1b*-hybridized embryo. The endothelial cells of the PCV are still labelled (blue arrowhead), while the signal is completely absent from the DA territory (red arrowhead). *prox1b* is now expressed in the space comprised between the DA and the PCV (arrow), where the TD forms starting from around 72 hpf. y, yolk. Scale bars represent 100 µm (A,B,C,D,G) or 20 µm (E,F,H,I).

*Prox1* is a master gene controlling the processes of budding, migration and proliferation of lymphangioblasts [2], [3], [4], [5], [6], [7]. In zebrafish, its contribution to lymphangiogenesis has been demonstrated by means of *prox1a* knockdown [4]. In this context, we examined the possible involvement of *prox1b* in lymphangiogenesis. The first signal of gene activity appeared in the central nervous system (CNS) starting from somitogenesis (10 s stage, Figure 2A), where *prox1b* expression persists during development (Figure 2B,C,D,G) (a more detailed analysis of *prox1b* mRNA distribution in the nervous system will be discussed elsewhere). Interestingly, at 48 hours post-fertilization (hpf), we detected intense *prox1b* staining at the level of the posterior cardinal vein (PCV) and of the sprouts emanating from this vessel into the intersegmental space (Figure 2D,E). The analysis of transversal sections of hybridized embryos revealed that *prox1b* is indeed expressed in the endothelial cells of the vein wall (Figure 2F, bottom), from where lymphatic precursor emerge to migrate towards the horizontal myoseptum region. Later on, according to previous studies, these lymphatic precursors will migrate from the horizontal myoseptum to a position ventral to the dorsal aorta (DA) and contribute to the mature TD [4], [14]. Notably, we observed the expression of *prox1b* on both sides of the trunk, adjacent to the notochord, at the level of the horizontal myoseptum (Figure 2F, top). Also interesting, the wall of the DA was not marked by *prox1b* expression (Figure 2F, bottom). At 72 hpf *prox1b* signal was still visible at the level of the intersomitic boundaries (Figure 2G,H) and the PCV (Figure 2G,H,I), and appeared in the space comprised between the DA and the PCV (Figure 2I), where the TD forms starting from around 3 days post-fertilization (dpf) [4], [14]. It is noteworthy that *prox1b* medaka ortholog mRNA was not detectable until 3 dpf (34 somite stage) and could be seen exclusively in few territories of the CNS during development [9].

**Figure 3:**
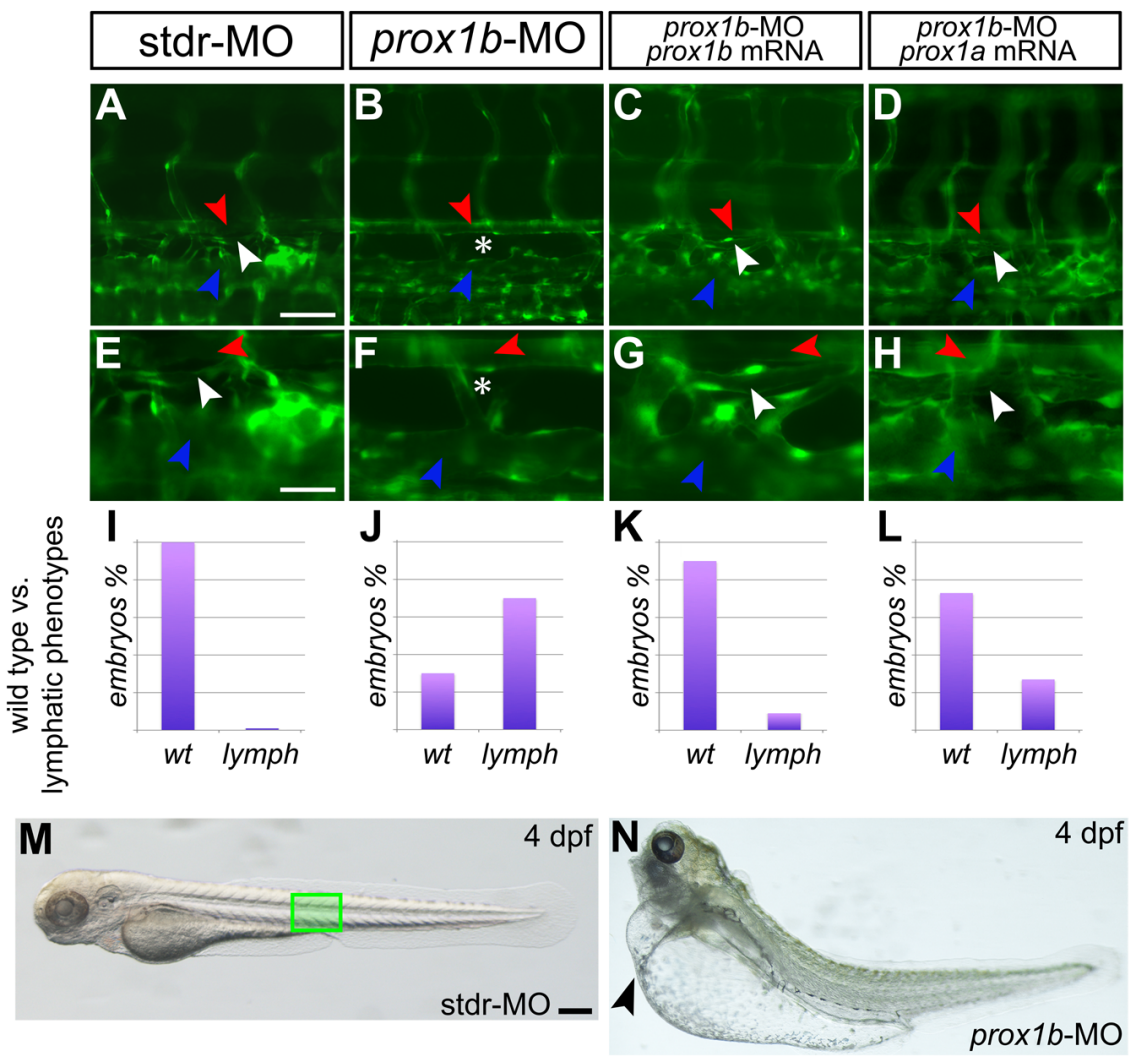
*prox1b* depletion results in the complete loss of the thoracic duct. fli1:GFP expression labels the DA (red arrowheads), the PCV (blue arrowheads), and lymphatic TD (white arrowheads) in 5 dpf embryos. A–D are shown magnified below in E–H. In comparison to control embryos injected with the stdr-MO (A,E), where 100% of the embryos analyzed displays a normal TD (I), the MO directed against *prox1b* (B,F) results in the absence of the TD (asterisks) in 70% of injected embryos (J). (C,G) the specificity of the effect of the *prox1b*-MO is confirmed by the ability of *prox1b* mRNA to rescue the lymphatic phenotype. (K) indeed, MO/mRNA coinjection resulted in about 90% of the embryos showing a normal TD. (D,H) *prox1a* mRNA is able to rescue the lymphatic phenotype induced by *prox1b*-MO injection in about 75% of the embryos analyzed (L). (M) 4 dpf control (stdr-MO) injected embryos compared to the (N) *prox1b* morphant (*prox1b*-MO) at the same developmental stage displaying severe edema (arrowhead). The green box in M indicates the approximate position of the embryo trunk depicted in A–D. Scale bars represent 40 µm (A,B,C,D), 20 µm (E,F,G,H), or 100 µm (M,N).

**Figure 4:**
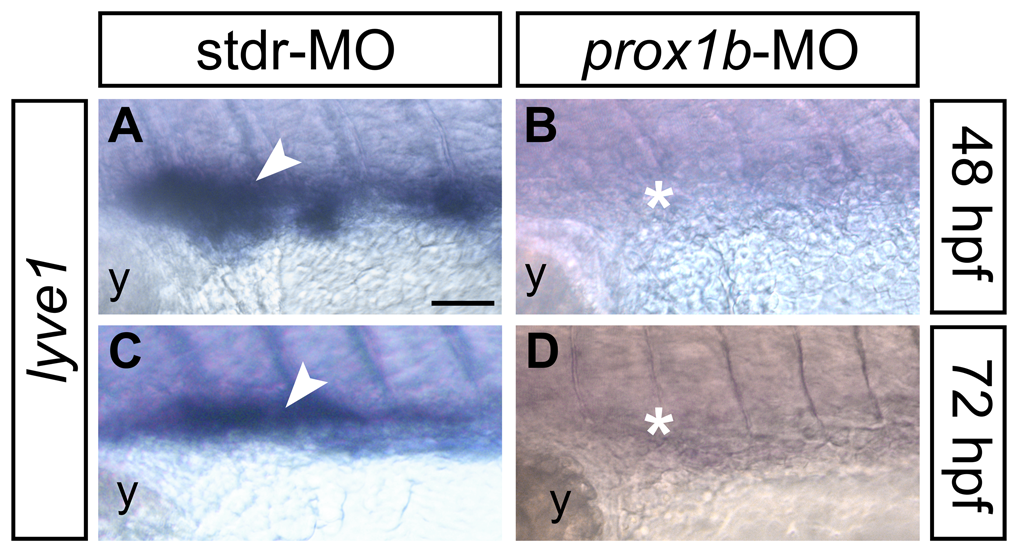
The expression of the lymphatic molecular marker *lyve1* is dramatically reduced in *prox1b* morphants. *lyve1* riboprobe labels developing lymphatic endothelial cells in 48 (A,B), and 72 (C,D) hpf embryos (all lateral views, anterior to the left; the approximate region of the embryo trunk imaged in all panels corresponds to the green box in Figure 3M). In comparison to control embryos injected with the stdr-MO (A,C), that display a normal expression of *lyve1* (white arrowheads), the MO directed against *prox1b* (B,D) results in the absence of *lyve1* signal (white asterisks). y, yolk. Scale bar represents 40 µm.

The pattern of expression of zebrafish *prox1b* led us to investigate the possible functional role of the gene in lymphangiogenesis. *prox1b* knockdown produced a lymphatic phenotypes that we evaluated searching for the presence/absence of the TD in fli1:GFP transgenic fish [15] at 5 dpf (Figure 3), the developmental stage at which the TD is already fully formed [4], [10]. In comparison to control embryos (Figure 3A,E,I), *prox1b* knockdown resulted in the absence of the TD (Figure 3B,F) in 70% of injected embryos (n = 70) (Figure 3J). Interestingly, the lack of the TD determined by the injection of *prox1b*-MO was always associated to mild to severe cardiac edema (Figure 3M,N), resembling the *prox1a*-lymphatic phenotype previously described [4]. A small percentage of the injected embryos displayed impaired circulation, and heart and trunk defects that did not allowed the observation of the vasculature, and for this reason was not included in the final analysis. The specificity of the lymphatic phenotype induced by *prox1b* knockdown was verified through the coinjection of a *prox1b* full-length mRNA properly mutagenized in the region targeted by the morpholino, which rescued the normal phenotype (Fig 3C,G,K). In order to corroborate the role of *prox1b* in lymphangiogenesis, the expression of *lyve1*, a specific molecular marker for lymphatic endothelial cells [14], [16], has been assayed (Figure 4). In comparison to control embryos (Figure 4A,C), *lyve1* expression in the trunk of *prox1b* morphants was weak or completely absent at 48 (80%, n = 30; Figure 4B) and 72 hpf (82%, n = 25; Figure 4D), indicating an impairment of lymphatic development.

To ascertain whether *prox1b* might synergize with *prox1a* to promote lymphangiogenesis, we coinjected the two orthologs specific MOs. The simultaneous knockdown of the two genes did not determine more severe phenotypes or the increase of the number of affected embryos in comparison to the *prox1b*-MO single morphants (data not shown). The attempt to use higher MOs concentrations resulted in acute generalized defects that impeded additional analysis of the morphants, as previously reported for the single *prox1a*–MO injection [10]. However, it is remarkable to notice that the *prox1b*-induced lymphatic phenotype could be rescued by means of coinjection with *prox1a* mRNA. Indeed, 75% of the double injected embryos (n = 75) appeared phenotipically normal with a perfectly formed TD (Figure 3D,H,L), highlighting the evolutionary conservation between the two orthologs.

Altogether these data support the indispensable role of *prox1b* in zebrafish lymphangiogenesis, representing a contribution of basic importance for the consolidation of zebrafish as a valuable lymphangiogenic model.

## Methods

### Ethics Statement

All embryos were handled according to relevant national and international guidelines.

### Fish and Embryos Maintenance

Fish of the AB strain and transgenics for fli:GFP [15] were maintained at 28°C on a 14-hr light/10-hr dark cycle. Embryos were collected by natural spawning and staged according to Kimmel and colleagues [17].

### prox1b identification and cDNA cloning

Zebrafish *prox1b* gene was identified through *in-silico* search using *prox1a* full-length cDNA as a bait. Two gene specific primers (*prox1b*F: 5′-CACCGCCATATAACACCACA-3′, and *prox1b*R: 5′-TTAACTGCTGGCCCTCCTGT-3′) have been used to amplify the full-length cDNA. The 3′ UTR have been obtained through RACE technique while the 5′ UTR has been identified searching the GenBank ESTs database.

### Whole mount in-situ hybridization (WISH)

WISH was carried out as previously described [18], [19]. Histological analysis of previously hybridized embryos was carried out on 8 µm sections.

### Injections

Injections were carried out on 1- to 2-cell stage embryos. To repress *prox1b* mRNAs translation, an ATG-targeting morpholino (MO) was synthesized (Gene Tools): 5′-GGGAATCCATAGCCTCCTTTTCTGT-3′. *prox1b*-specific MO was used at the concentration of 6 ng per embryo in 1X Danieau buffer (pH 7,6). To repress *prox1a* mRNA translation, a *prox1a*-specific MO was used at the concentration of 8 ng per embryo, as previously reported [4], [12], [20], [21]. As control, we injected 8 ng per embryo of a standard control MO (stdr-MO). MO-mRNA double injection experiments were conducted using 300 pg of capped mRNA per embryo together with the above reported amount of the MO of interest. For live microscopy observation, 5 dpf fli1:GFP transgenic larvae have been anesthetized and monitored under a fluorescent microscope. For the *in-vivo* test of the specificity of *prox1b*-MO, 300 pg per embryo of the *prox1b*-GFP sensor plasmid have been coinjected with 8 ng of *prox1b*-MO or stdr-MO, respectively (Figure S1). The presence/absence of the GFP signal has been monitored under a fluorescent microscope from 24 to 48 hpf.

## Supporting Information

Figure S1 prox1b-MO specifically reduces prox1b mRNA translation. For the in-vivo test of the specificity of prox1b-MO, a prox1b-GFP sensor has been generated. (A) The construct contains 96 bp of the 5′ UTR, and the first 432 bp of the prox1b coding sequence (Nprox1b) fused with the GFP open reading frame. The blue bar indicates the region of the mRNA targeted by the prox1b-MO. The construct, obtained by PCR, has been cloned into the pTargeT expression vector (Promega) and used for injection experiments. (B) GFP-positive cells in the trunk (inset and arrowheads) and (C) in the yolk epithelium (arrowheads) are visible following coinjection of the sensor and the stdr-MO. (D) The complete absence of fusion protein expression when the sensor is coinjected with prox1b-MO confirms the specificity of action of the morpholino. Scale bar represents 100 µm.
(6.42 MB TIF)
