# Correction: *prox1b* Activity Is Essential in Zebrafish Lymphangiogenesis

**DOI:** 10.1371/annotation/6c1f2c4a-7da6-43cd-a934-78b7550dfbaf

**Published:** 2010-11-03

**Authors:** Luca Del Giacco, Anna Pistocchi, Anna Ghilardi

The first author's name is incorrect in the citation. The correct citation is Del Giacco L. 

