# Supplementary figures and images for: prox1b Activity Is Essential in Zebrafish Lymphangiogenesis
Source: PLoS One. 2010 Oct 18;5(10):e13170. doi: 10.1371/journal.pone.0013170 (PMC2956630; doi:10.1371/journal.pone.0013170)

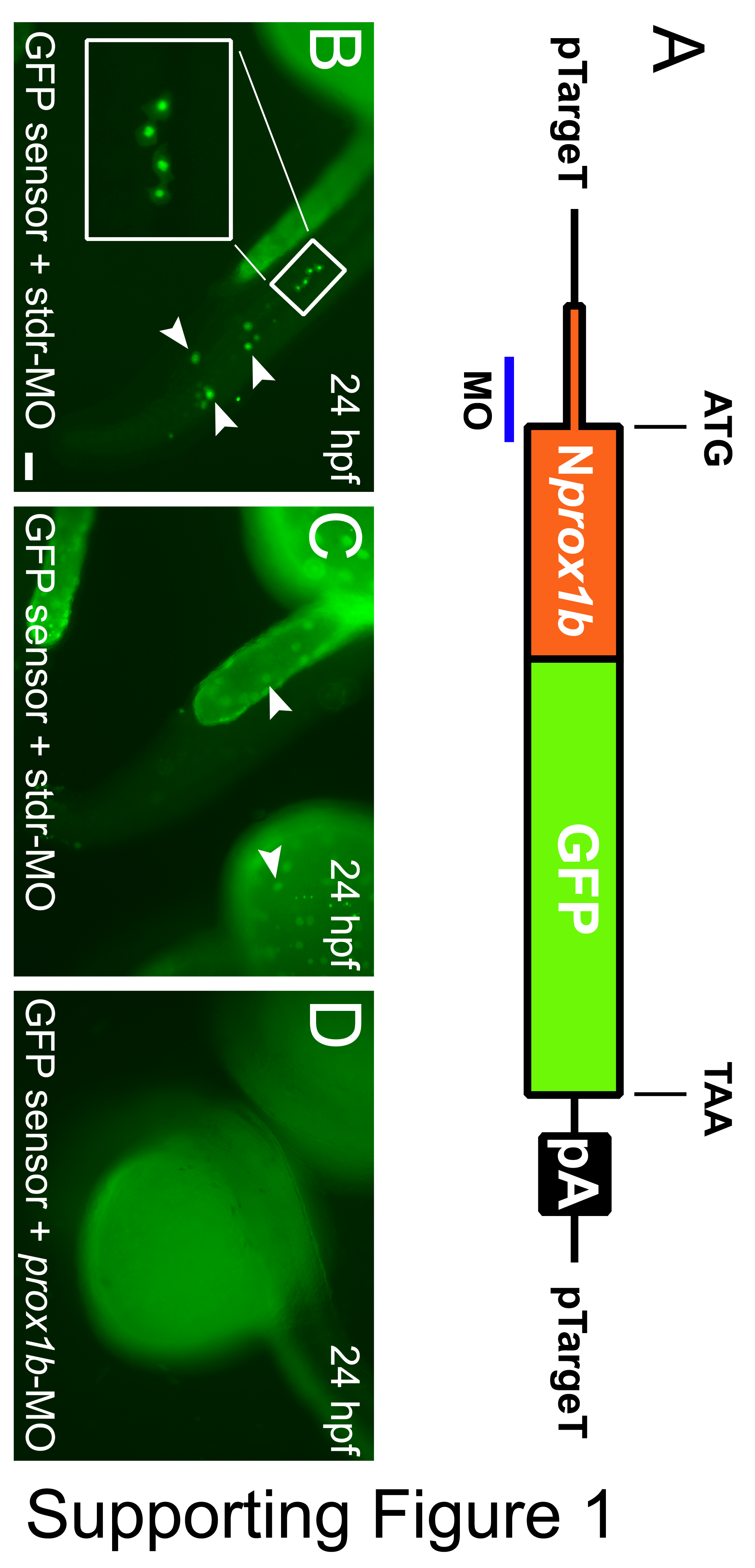

Supplement: Figure S1 — prox1b-MO specifically reduces prox1b mRNA translation. For the in-vivo test of the specificity of prox1b-MO, a prox1b-GFP sensor has been generated. (A) The construct contains 96 bp of the 5′ UTR, and the first 432 bp of the prox1b coding sequence (Nprox1b) fused with the GFP open reading frame. The blue bar indicates the region of the mRNA targeted by the prox1b-MO. The construct, obtained by PCR, has been cloned into the pTargeT expression vector (Promega) and used for injection experiments. (B) GFP-positive cells in the trunk (inset and arrowheads) and (C) in the yolk epithelium (arrowheads) are visible following coinjection of the sensor and the stdr-MO. (D) The complete absence of fusion protein expression when the sensor is coinjected with prox1b-MO confirms the specificity of action of the morpholino. Scale bar represents 100 µm. (6.42 MB TIF) [file pone.0013170.s001.tif]
